# Supplementary material for: Measurement of hindered diffusion in complex geometries for high-speed studies of single-molecule forces
Source: Sci Rep. 2021 Jan 26;11:2196. doi: 10.1038/s41598-021-81593-x (PMC7838191; doi:10.1038/s41598-021-81593-x)
Supplement: Supplementary file 1 — Supplementary Information. [file 41598_2021_81593_MOESM1_ESM.docx]

Supplementary information for:

**Measurement of hindered diffusion in complex geometries
for high-speed studies of single-molecule forces**

Tobias F. Bartsch, Camila M. Villasante, Ahmed Touré, Daniel M. Firester, Felicitas E. Hengel, Aaron Oswald, and A. J. Hudspeth

Howard Hughes Medical Institute and Laboratory of Sensory Neuroscience, The Rockefeller University, New York, NY 10065 USA

# **Contents**

Supplementary Information Materials and Methods

Supplementary Information Figures and captions

Supplementary Information References

# **Supplementary Information Materials and Methods**

# *Preparation of stationary pedestal beads*

Sample preparation began with the covalent attachment of pedestal beads to a functionalized coverslip. Aminated 2 µm silicon dioxide microspheres (140414‑10, Corpuscular, Cold Spring, New York, USA), which served as pedestal beads, were first conjugated to Cys-SpyCatcher molecules (EOX004, Kerafast, Boston, MA, USA) through *N*‑hydroxysuccinimide-PEG12‑maleimide spacers (22112, ThermoFisher, Waltham, Ma, USA). In order to efficiently couple the pedestal beads and polyethylene glycol spacers, the surface amine groups of the pedestal beads were first deprotonated. This was achieved by washing 200 µL of the beads, and then resuspending the beads in 100 µl of 50 mM sodium tetraborate buffer (11625, Sigma Aldrich, St Louis, MO, USA), pH 8.5, for 1 hr at room temperature. The spacer was added to the bead solution to achieve a 50 mM concentration of the spacer in a volume of 160 µL. The mixture was incubated for 30 min at room temperature, washed three times with Hepes-buffered saline solution (HeBS; 20 mM Hepes and 100 mM NaCl), and resuspended into 250 µL HeBs. Concurrently, 0.5 mg of Cys-SpyCatcher protein was dissolved in 50 µL of HeBs and reduced with tris(2‑carboxyethyl)phosphine (Immobilized TCEP Disulfide Reducing Gel, 77712, ThermoFisher Scientific, Waltham, MA, USA) for 1 hr. The Cys-SpyCatcher protein solution was then mixed with 55 µL of 100 mg/mL sulfhydryl-blocked bovine serum albumin (BSA; 100‑10SB, Lee Biosolutions, Inc., Maryland Heights, MO, USA) in HeBS. The pedestal bead solution was then added to the Cys-SpyCatcher solution, and the resulting mixture was incubated overnight at 4 °C. The beads were washed three times with 1 mL HeBs and resuspended in 100 µL HeBs. Unreacted maleimide was quenched by mixing the bead solution with 100 µL of 1 M L-cysteine (11033-016, Gibco BRL, Gaithersburg, MD, USA) in HeBS and incubating for 1 hr. The pedestal beads were washed three times and stored in HeBs at 4 ˚C.

# *Preparation of coverslips*

Glass coverslips (12-545-81, Thermo Fisher Scientific, Waltham, MA, USA) were functionalized in order to enable covalent bonding with pedestal beads. The coverslips were first cleaned through sonication in ethanol for 15 min, then dried with oxygen gas. The coverslips were placed in a mixture of 1.5 g Nochromix (Godax Laboratories, Cabin John, MD, USA) in 60 mL sulfuric acid (A300S-500, Thermo Fisher Scientific, Waltham, MA, USA) for 3 hr. The coverslips were then washed in six 5 min sonication steps, three times in ethanol and then three times in deionized water. After drying with oxygen gas, the slides were oxidized in an ultraviolet ozone cleaner (PC440, Bioforce Nanosciences, Salt Lake City, UT, USA). To allow vapor deposition of an aminosilane layer, the coverslips were placed overnight at 80 °C in an air-tight glass container with 100 µL 5‑(3-aminopropyl)trimethoxysilane (281778, Sigma Aldrich, Inc, St Louis, MO, USA) dissolved in 10 mL of toluene (T324-1, Thermo Fisher Scientific, Waltham, MA, USA). The coverslips were washed with ethanol and dried with oxygen gas. To convert the amine groups from the aminosilane coating into carboxyl groups, a solution of 50 mg succinic anhydride (S7626, Sigma Aldrich, St Louis, MO, USA) dissolved in 1 mL dimethyl sulfoxide (D4540, Sigma Aldrich, St Louis, MO, USA) was placed on the coverslips, which were incubated for 3 hr at room temperature. The coverslips were then washed three times with ethanol and dried with oxygen.

# *Preparation of experimental chambers*

A coverslip was mounted to a metal washer using vacuum grease, creating a rectangular well with the carboxylated surface exposed. In order to functionalize the coverslip with amine-reactive hydroxysulfosuccinimide esters, 10 mg of (1‑ethyl-3‑(3‑dimethylaminopropyl)carbodiimide (77149, Thermo Fisher Scientific, Waltham, MA, USA) and 10 mg of N‑hydroxysulfosuccinimide (24510, Thermo Fisher Scientific, Waltham, MA, USA) were dissolved in 1 mL of activation buffer containing 10 mM NaCl and 1 mM 2‑(N‑morpholino)ethanesulfonic acid (M3671, Sigma Aldrich, St Louis, MO, USA) at pH 6; 50 µL of the resulting solution was then added to the carboxylated surface of the mounted coverslip. After washing the functionalized surface with 2 mL Hepes-buffered saline solution with Ca^2+^, we added the bead solution to the surface and allowed the reaction to proceed for 2 hr. The addition of a second coverslip enclosed the sample chamber except for a pair of access ports to allow liquid exchange. The liquid in the chamber was replaced with blocking-buffer solution containing 10 mg/mL sulfhydryl-blocked bovine serum albumin (100‑10SB, Lee Biosolutions, Inc., Maryland Heights, MO, USA), 150 mM NaCl, 20 mM Tris‑HCl, and 3 mM CaCl2 at pH 8. The chamber was then incubated overnight at 4 ˚C.

# *Addition of diffusing probe beads*

Streptavidin-coated polystyrene microspheres 1 µm in diameter (CP01004, Bangs Laboratories, Fishers, IN, USA), which served as probe beads, were added to the sample chamber in a solution containing 20 mM Tris‑HCl at pH 7.5, 150 mM NaCl, 3 mM Ca^2+^ and 10 mg/mL sulfhydryl-blocked bovine serum albumin (100‑10SB, Lee Biosolutions, Inc., Maryland Heights, MO, USA). The experiments with PCDH15 instead involved a Ca^2+^ concentration of 20 μM. For thermal noise imaging experiments involving tethered peptide, peptide was diluted and added to the sample for 1 hr at 4 ˚C prior to the addition of probe beads. After washing the sample with blocking buffer solution to remove peptide unbound to the pedestal bead, we then added probe beads. The peptide was a fusion of two sequences used as linkers in PCDH15 experiments. The first sequence, SpyTag (**AHIVMVDAYKPTK**), forms covalent bonds with SpyCatcher proteins on the pedestal bead surface. SpyTag was connected to a second sequence, AviTag **(GLNDIFEAQKIEWHE)**, through a **GGGSGGGS** spacer. The AviTag component was then biotinylated by biotin ligase (BirA‑500, Avidity, Aurora, CO, USA), permitting interaction with probe beads through their streptavidin coating.

# *Optical trap*

The photonic-force microscope used in these experiments was capable of measuring the position of a micrometer-sized probe bead with an integration time of 1 µs, sampled at 10 µs intervals, with sub-nanometer precision. A weak optical trap was formed within the sample chamber by focusing a 1064 nm laser beam with a high numerical-aperture water-immersion objective lens (Figure SI1). In 81 experiments, the trap's stiffness averaged 3.9 ± 1.0 μN·m^‑1^ along the *x*‑axis, 4.6 ± 1.1 μN·m^‑1^ along the *y*‑axis, and 1.1 ± 0.2 μN·m^‑1^ along the *z*‑axis (means ± standard deviations). The three-dimensional position of the probe confined within the weak optical trap was obtained from the interference on a quadrant photodiode of light scattered forward from the probe with unscattered light.

To conduct an experiment, we mounted the sample chamber on a nano-positioning stage (NanoView/M375HS, Mad City Labs, WI, USA). The immediate area of the sample surrounding the optical traps was visualized with a camera that received brightfield images of the sample from illumination by a light-emitting diode. The location of the optical trap within the sample could be adjusted by changing the three-dimensional position of the nano-positioning stage. This arrangement also permitted adjustment of a pedestal bead’s position with respect to the trap.

Freely diffusing probe beads were also trapped in this manner. To hold the probe at a constant displacement from the center of weak optical trap, as was required for the correction owing to the pedestal, a stimulus trap was formed by an 852 nm laser. The position of this relatively strong optical trap with respect to the weak trap was adjusted by means of a beam-steering lens mounted on a three-dimensional piezoelectric-block translator in the beam path of the strong laser.

# *Electrostatic interactions between beads*

Although electrostatic interactions between the pedestal and probe beads might affect our measurements, three considerations render this effect minor. First, the polystyrene probe beads are essentially uncharged. Next, our experiments were conducted in the presence of bovine serum albumin, which coats the beads and largely counters any surface charge. And finally, the moderately concentrated ionic solution restricted electrostatic effects to a distance of a few nanometers. The Debye screening length is given by

$$\lambda_{D}=\frac{1}{\kappa}=\sqrt{\frac{\varepsilon\varepsilon_{0}k_{B}T}{N_{A}e^{2}\sum C_{i}z_{i}^{2}} ,}$$

in which *ε* is the dielectric constant of the aqueous medium, *ε*_0_ is the vacuum permittivity, *k*_B_ is the Boltzmann constant, *T* is the thermodynamic temperature, *N*_A_ is the Avogadro number, *e* is the elementary charge, and *C*_i_ and *z*_i_ are respectively the concentration (in moles per cubic meter) and charge of the ions. Considering only the chief component of the solution used in these experiments, 150 mM NaCl, we find that λ_D_ ≈ 7.9 nm. The additional 20 mM of Tris·HCl and 3 mM CaCl_2_ provide additional screening, especially inasmuch as the divalent ion contributes according to the square of its concentration: we estimate that λ_D_ ≈ 7.4 nm during experiments. The effects of any surface charges would accordingly decline to less than 5 % over about 20 nm. As a consequence, surface charge might affect only the data points for the closest apposition of the beads.

# *Choice of sampling intervals*

The measured diffusion constant of a spherical particle undergoing Brownian motion in an optical trap depends both on the starting position of the bead and on the choice of time lag. In our experimental findings, we determined the position-dependent and anisotropic diffusion constant *D* of a 1 µm probe bead by partitioning the trapping volume into voxels and then measuring the displacement along each axis of a bead that originated within a given voxel. Under free-diffusive conditions, the mean squared displacement of a particle scales linearly with time, with a slope given by 2*D* [**1,2**]. This formula permits calculation of the local diffusion constant, coarse-grained over the voxel partitioning.

The presence of an additional external force, owing to the optical trap itself, complicates analysis and introduces the possibility of super- or sub-diffusive behavior on short timescales. To better understand of these effects, we considered the motion of a diffusing bead in an optical trap as an Ornstein-Uhlenbeck process in the absence of a drift term [**3**]; the optical trapping force acts to confine the bead and the random thermal force drives Brownian motion. The equation of motion of the optically trapped bead is given by the stochastic Langevin equation:

$\gamma_{i}\frac{dx_{i}}{dt}=-\kappa_{i}x_{i}+\xi_{i}$ (S1)

in which the indices represent independent *x*‑, *y*‑, and *z*‑directions, γ is the local viscous drag coefficient of the probe given by Stokes’ law, *x* is the time-dependent position of the bead with respect to the center of the trap, κ is the effective spring constant of the optical trap, and ξ is a random thermal force with first and second moments given by $\left\langle\xi(t) \right\rangle=0$ and $\left\langle\xi(t)\xi(t^{'}) \right\rangle=2\gamma k_{B}T\delta(t-t^{'})$ [**4**]. To simplify our analysis, we assume the diffusion constant remains invariant over the course of a single measurement; that is, it depends only on the starting voxel and axis of measurement. This approximation is valid so long as the time lag is small, but can fail for larger time lags during which the bead samples large portions of the trapping volume. Although the following analysis neglects the effects of the hindered diffusion that we have measured, in all cases this simplifying first-order approximation results in an overly conservative estimate of the drag, and accordingly underestimation of the timescale at which deviations from free diffusion occur.

With this simplifying assumption, the formal solution of the Ornstein-Uhlenbeck process given in Equation S1 is:

$x\left( t \right)=ae^{-\frac{\kappa}{\gamma}t}+\sqrt{{2k_{B}T}/\gamma}\int_{0}^{t} e^{-\frac{\kappa}{\gamma}(t-t^{'})}dt'$ (S2)

in which *a* is the starting displacement of the bead from the center of the optical trap and we adopt the Itō interpretation of the stochastic integral [**5]**. Although each measured *x*(*t*) is dependent on a particular realization of the Wiener process, the moments of *x*(*t*) are nevertheless well defined. By definition, the mean squared displacement is given by $MSD=\left\langle\left( x\left( t \right)-a \right)^{2} \right\rangle$, which can be solved explicitly using Equation S2:

$MSD=\frac{k_{B}T}{\kappa}\left( 1-e^{-\frac{2\kappa}{\gamma}t} \right)+a^{2}\left( 1+e^{-\frac{2\kappa}{\gamma}t}-2e^{-\frac{\kappa}{\gamma}t} \right)$ (S3)

Here we make use of the fact that the expected value of an Itô integral is zero, and used the Itō isometry to determine the higher-order moments [**5**]. If the starting position of the bead is zero, the equation reduces to the familiar variance of a particle undergoing diffusion in a harmonic potential. Under this condition, the bead transitions from free diffusion at timescales much less than the characteristic autocorrelation time of trapping $\tau=\gamma/{2\kappa}$ to confined sub-diffusive behavior at later times (Figure SI2).

Further complications arise for measurements of local diffusion constants in voxels far from the center of the trap, for which both terms of Equation S3 must be considered. For sufficiently distant starting positions, the gradient force on the bead may cause various degrees of super-diffusive motion, up to ballistic motion under certain conditions. In the limit of *t* << τ, the mean squared displacement may be approximated as

$MSD=\frac{2k_{B}T}{\gamma}t+\frac{4\kappa^{2}a^{2}}{\gamma^{2}}t^{2}$ (S4)

which reduces to free diffusion for sufficiently small choice of time lag, but may become super-diffusive at intermediate time lags.

To better understand the effects of time lag on our measurements, we considered the ratio of the naively measured diffusion constant to the true free-diffusion constant, $R_{D}={D_{M}}/{D_{F}}$, which reflects either an overestimate or an underestimate of the local diffusion constant. The naively measured diffusion constant was extracted from the equation $MSD=2D_{M}t$ in the presence of the optical trap, whereas the true free diffusion constant was defined through the Einstein relation $\gamma D=k_{B}T$.

For time lags much less than the characteristic autocorrelation time, the ratio *R*_D_ remained close to unity, whereas for intermediate time lags it deviated significantly as a result either of the gradient force or of confinement by the optical trap. Decreasing the trapping stiffness results in similar but diminished effects (Figures SI3 and SI4).

Finally, we quantified the earliest time lag at which the measured diffusion constant was expected to deviate more than 10 % from that for free diffusion. Along the *x*‑ and *y*‑axes, for which the spring constant of the optical trap was near 4 µN·m^‑1^, a time lag of 150 µs was determined to be sufficiently short if the voxel under consideration was within 65 nm of the trap's center. Along the *z*‑axis, for which the spring constant was approximately 1 µN·m^‑1^, the experimentally chosen time lag was sufficiently short to ensure accurate estimation of the free diffusion coefficient within 150 nm of the trap's center (Figure SI5).

# *Data presentation*

To generate plots of the experimental data, we used Igor Pro (version 8.04, WaveMetrics, Lake Oswego, OR, USA). The Mayavi [**6**] Python application (version 4.7.2) was used to create the heat maps.

# **Supplementary Information Figures and captions**

**
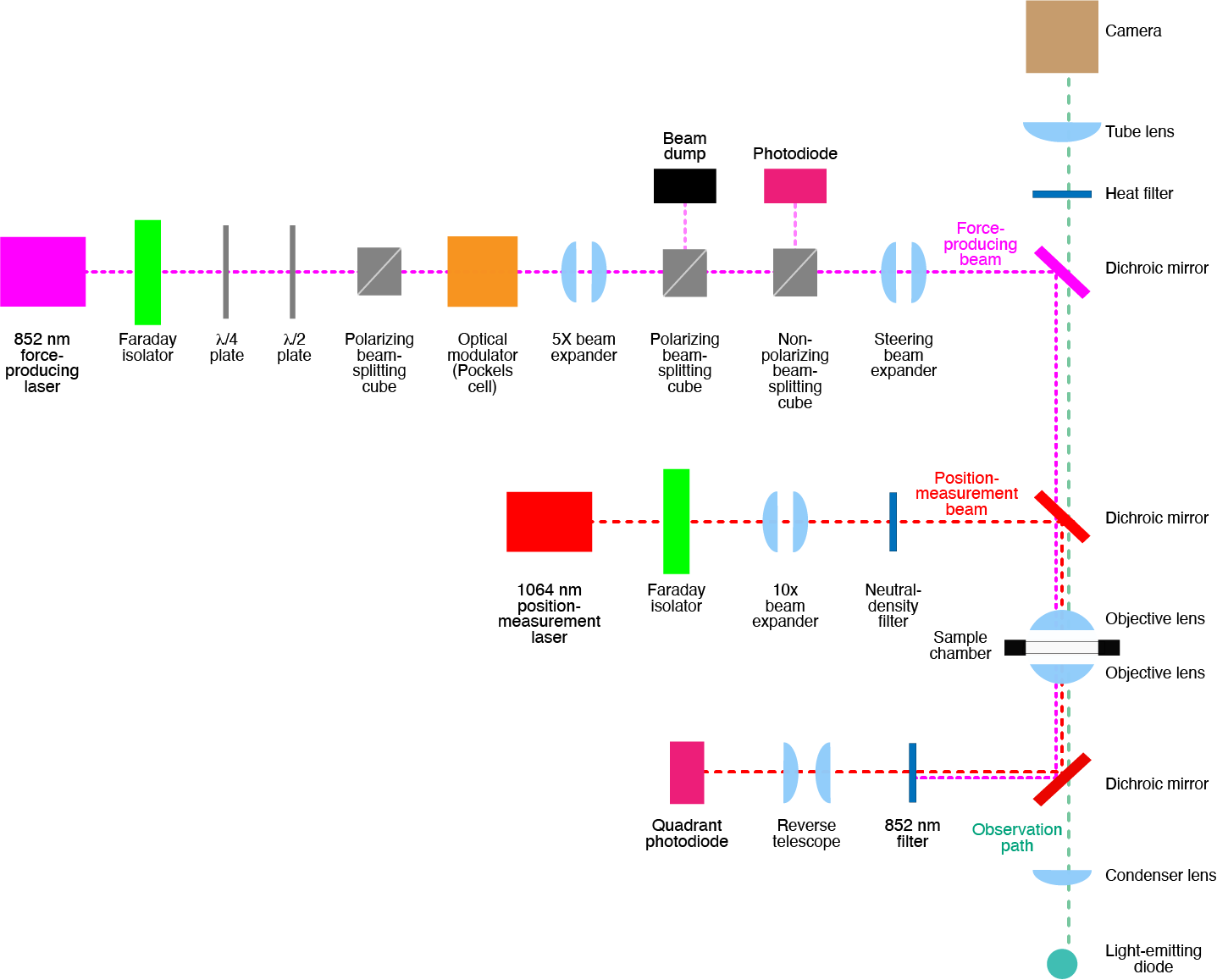
**

**Figure SI1. Configuration of the experimental apparatus.** The system includes three light paths that have been offset laterally in the schematic diagram for the sake of clarity. (1) The force-producing beam (fuchsia), which originates from a 400 mW 852 nm laser (DL852‑400, CrystaLaser, Reno, NV, USA), first passes through a Faraday isolator (IO‑3D‑850‑VLP, Thorlabs Inc., Newton, NJ, USA) to prevent light from being reflected back into the laser. The light traverses two wave plates to polarize the beam in the perpendicular direction before reaching a polarizing beam-splitting cube that removes any light that is incorrectly polarized. A Pockels cell (Laser Modulators LM 13, 8450‑202‑301‑5, Qioptiq, Feldkirchen, Germany) permits adjustment of the beam's intensity and thus of the holding force acting on the probe bead. After passing through a beam expander (GBE05‑B, Thorlabs), the beam is cleaned with another polarizing beam-splitting cube that directs inappropriately polarized light into a dump. A non-polarizing beamsplitting cube reflects 10 % of the beam to a photodiode (Öffner MSR Technik, Plankstadt, Germany) that measures the laser's power and allows determination of the optical trap's spring constant. After passing through a shutter, the beam reaches a beam steering lens that allows positioning in the plane of the specimen with a piezoeletrical nanopositioning stage (P‑282.30, Physik Instrumente GmbH, Karlsruhe, Germany). A dichroic mirror (ZT775sp‑2p‑UF3, Chroma Technology Corporation, Bellow Falls, VT, USA) then directs the beam into the main optical path, where it traverses an upper objective lens and impinges upon the specimen. After exerting force on the probe bead, the beam is captured by a lower objective lens, reflected by a dichroic mirror, and intercepted by an 852 nm filter. (2) The position-measurement beam (red) from a 500 mW 1064 nm laser (Mephisto, Coherent, CA, USA) passes through a Faraday isolator, a beam expander (Sill Optics GmbH, Wendelstein, Germany), a neutral-density filter (Thorlabs) to attenuate the power, and a shutter. A dichroic mirror then joins this beam with the 852 nm force-producing beam. After traversing the upper objective lens (OL UPSAPO 60XW, NA 1.2, Olympus Corporation, Tokyo, Japan), the specimen, and the lower objective lens (LUMPLFLN 60XW, Olympus), the position-sensing beam is reflected from a dichroic mirror and passed through a filter (FF01‑937/LP‑25, Semrock Inc., Rochester, NY, USA) that transmits only light from the 1064 nm laser. After passing through a reverse telescope to reduce its size, the beam impinges on a quadrant photodiode (Öffner MSR Technik, Plankstadt, Germany). (3) The observation path (teal) originates from a light-emitting diode, passes through a condenser lens, and enters the main optical path. After traversing the objective lenses and specimen, the light is transmitted by the dichroic mirrors associated with the two laser-beam paths and is focussed by a tube lens (Achromate VIS, G322246000, Qioptiq) onto the active surface of the water-cooled CMOS camera (pco.edge 5.5, PCO‑TECH Inc., Romulus, MI, USA).

**
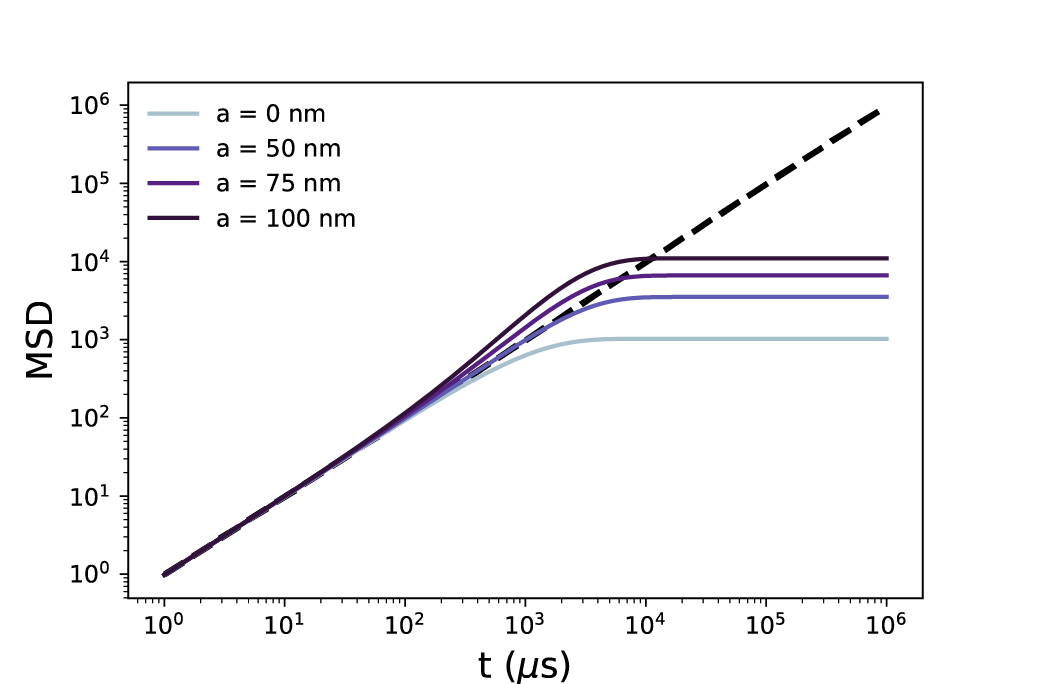
**

**Figure SI2. Mean squared displacements in an optical trap.** The plots show solutions to Equation SI3 for κ = 4 µN·m^‑1^, γ = 8.4 nN·s·m^‑1^, and *k*_B_*T* = 4.11 zJ and for starting displacements of *a* = 0 nm, 50 nm, 75 nm, and 100 nm. In dashed black, the theoretical free diffusion limit. For starting displacements close to the center of the trap, the bead undergoes standard diffusion before becoming sub-diffusive and reaching a plateau due to trap confinement. Increasing the value of *a* initially increases the free-diffusion regime before causing super-diffusive behavior at intermediate time lags. High values of *a* correspond to mean squared displacements with regimes of both super-diffusive and sub-diffusive behavior.

**
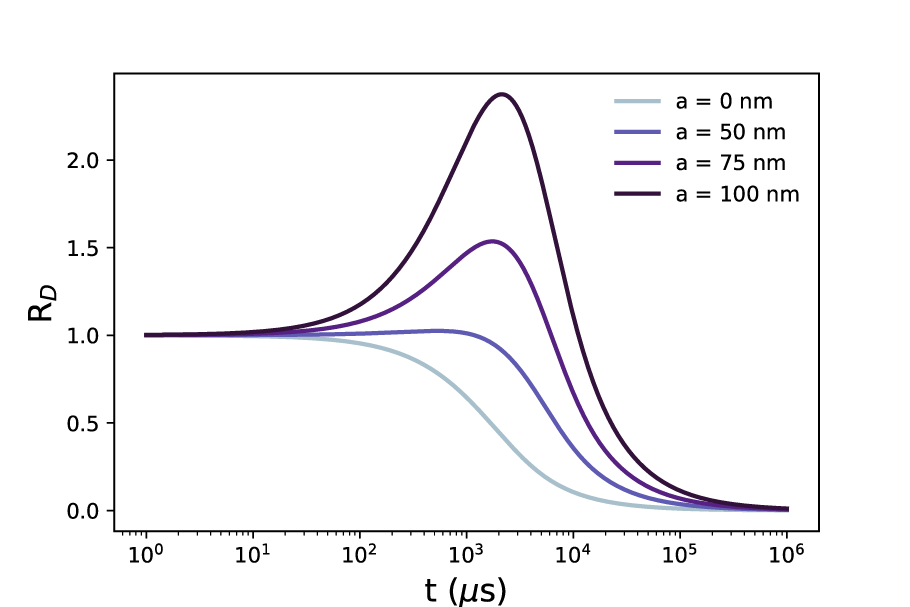
**

**Figure SI3. Ratio of measured to theoretical diffusion constants along the *x*‑ and *y*‑axes.** The naively measured diffusion constant, in the absence of consideration for an external trapping potential, can deviate significantly from the true diffusion constant that would be measured in the absence of any external potential. The ratio of the naively measured diffusion constant to the true diffusion constant is shown for an optical trapping spring constant of κ = 4 µN·m^‑1^ and a variety of starting positions *a*. For short time lags, the ratio remains close to unity for all values of *a*. At intermediate time lags, the ratio can deviate significantly from unity, resulting either in underestimation of the diffusion constant for voxels close to the center of the trap, or in overestimation of the diffusion constant for voxels far from the center of the trap. The regime over which the ratio remains close to unity extends significantly for *a* = 50 nm owing to cancellation of the trapping effect and gradient force.

**
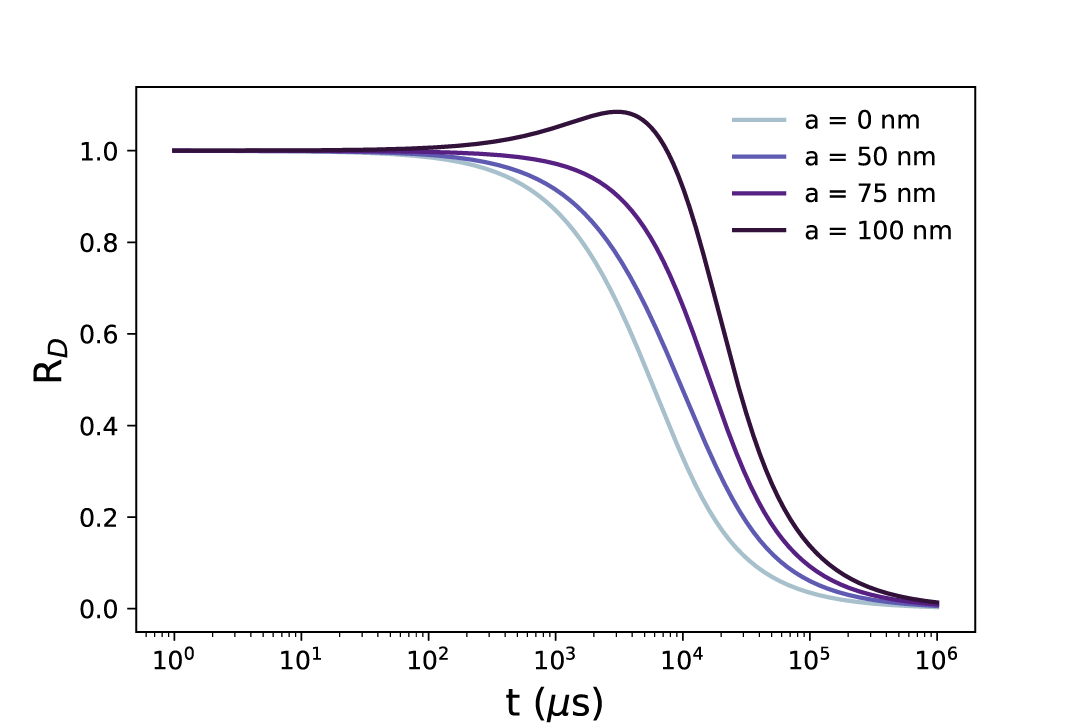
**

**Figure SI4. Ratio of measured diffusion constant to theoretical diffusion constant along the *z*‑axis.** The ratio of the naively measured diffusion constant to the true diffusion constant is shown for an optical trapping spring constant of κ = 1 µN·m^‑1^ and a variety of starting positions *a*. Because of the decreased spring constant along the *z*‑direction, only voxels far from the center of the trap exhibit super-diffusive behaviors.

**
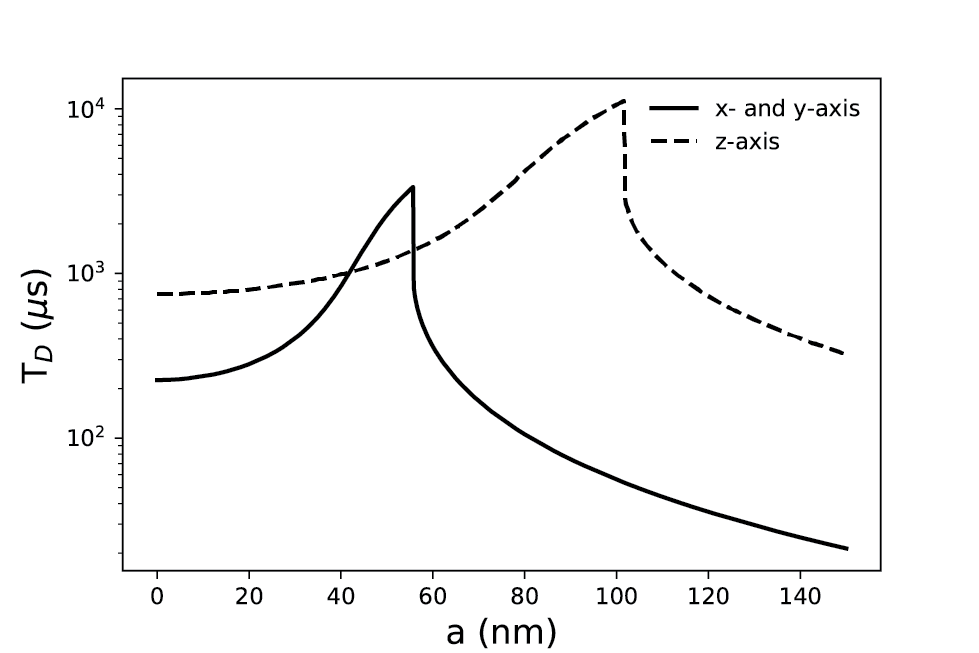
**

**Figure SI5. First times of deviation from freely diffusive behavior.** The figures show numerical solutions of the first deviation time *T*_D_ for γ = 8.4 nN·s·m^‑1^ and *k*_B_*T* = 4.11 zJ, with κ = 4 µN·m^‑1^ along the *x*‑ and *y*‑axes and κ = 1 µN·m^‑1^ along the *z*‑axis. The first deviation time was defined as the shortest time lag at which the naively measured diffusion constant deviated from the true diffusion constant by 10 %. Along the *x*‑ and *y*‑axes the first deviation times can become significantly lower than the experimentally chosen time lag of 150 µs for voxels far from the center of the trap. For all axes, the first deviation time increases as the starting position of the bead recedes from the center of the trap as a result of cancellation between the trapping and gradient forces. The time then falls abruptly owing to the super-diffusive effects of the gradient force.

# **Supplementary Information references**

1. Tischer, C., Pralle, A., and Florin, E.-L. (2004) Determination and correction of position detection nonlinearity in single particle tracking and three-dimensional scanning probe microscopy. *Microsc. Microanal*. **10:** 425–434.

2. Pralle, A., Florin, E.-L., Stelzer, E. H. K., and Hörber, J. K. H. (1998) Local viscosity probed by photonic force microscopy. *Appl. Phys. Mater. Sci. Process* **66:** S71–S73.

3. Uhlenbeck, G. E. and Ornstein, L. S. (1930) On the theory of Brownian motion. *Phys. Rev.* **36:** 823–841.

4. Nishi, K., Kilfoil, M., Schmidt, C., and MacKintosh, F. (2018) A symmetrical method to obtain shear moduli from microrheology. *Soft Matter* **14**, 10.1039/C7SM02499A.

5. Itō, K. (1944) Stochastic integral. *Proc. Imp. Acad.* **20:** 519–524.

6. Ramachandran, P. and Varoquaux, G. (2011) Mayavi: 3D visualization of scientific data. *IEEE Computing Sci. Engineer.* **13:** 40-51.
